# MADS-Box Transcription Factor *MadsA* Regulates Dimorphic Transition, Conidiation, and Germination of *Talaromyces marneffei*

**DOI:** 10.3389/fmicb.2018.01781

**Published:** 2018-08-07

**Authors:** Qiangyi Wang, Minghao Du, Shuai Wang, Linxia Liu, Liming Xiao, Linqi Wang, Tong Li, Hui Zhuang, Ence Yang

**Affiliations:** ^1^Department of Microbiology & Infectious Disease Center, School of Basic Medical Sciences, Peking University Health Science Center, Beijing, China; ^2^Institute of Systems Biomedicine, School of Basic Medical Sciences, Peking University Health Science Center, Beijing, China; ^3^Department of Laboratorial Science and Technology, School of Public Health, Peking University, Beijing, China; ^4^State Key Laboratory of Mycology, Institute of Microbiology, Chinese Academy of Sciences, Beijing, China; ^5^Institute of Microbiology, University of Chinese Academy of Sciences, Beijing, China

**Keywords:** *Talaromyces marneffei*, *madsA*, dimorphic transition, transcriptome, yeast-to-mycelium, oxidative stress

## Abstract

The opportunistic human pathogen *Talaromyces marneffei* exhibits a temperature-dependent dimorphic transition, which is closely related with its pathogenicity. This species grows as multinucleate mycelia that produce infectious conidia at 25°C, while undergoes a dimorphic transition to generate uninucleate yeast form cells at 37°C. The mechanisms of phenotype switching are not fully understood. The transcription factor *madsA* gene is a member of the MADS-box gene family. Previously, it was found that overexpression of *madsA* gene resulted in mycelial growth instead of yeast form at 37°C. In the current study, the *madsA* deletion mutant (Δ*madsA*) and complemented strain (CMA) were constructed by genetic manipulation. We compared the phenotypes, growth, conidiation, conidial germination and susceptibility to stresses (including osmotic and oxidative) of the Δ*madsA* with the wild-type (WT) and CMA strains. The results showed that the Δ*madsA* displayed a faster process of the yeast-to-mycelium transition than the WT and CMA. In addition, the deletion of *madsA* led to a delay in conidia production and conidial germination. The tolerance of Δ*madsA* conidia to hydrogen peroxide was better than that of the WT and CMA strains. Then, RNA-seq was performed to identify differences in gene expression between the Δ*madsA* mutant and WT strain during the yeast phase, mycelium phase, yeast-to-mycelium transition and mycelium-to-yeast transition, respectively. Gene ontology functional enrichment analyses indicated that some important processes such as transmembrane transport, oxidation-reduction process, protein catabolic process and response to oxidative stress were affected by the *madsA* deletion. Together, our results suggest that *madsA* functions as a global regulator involved in the conidiation and germination, especially in the dimorphic transition of *T. marneffei*. Its roles in the survival, pathogenicity and transmission of *T. marneffei* require further investigation.

## Introduction

*Talaromyces marneffei* (formerly *Penicillium marneffei*) is a significant emerging pathogenic fungus endemic in Southeast Asia and South China (Vanittanakom et al., 2006; Hu et al., 2013; Yilmaz et al., 2014). Although an increasing number of cases have been reported in non-human immunodeficiency virus (HIV) infected patients (Chan et al., 2016), *T. marneffei* mainly infects HIV-positive patients (Wong and Wong, 2011; Hu et al., 2013). In endemic areas, penicilliosis marneffei, the disease caused by *T. marneffei*, has been the third most prevalent opportunistic infection in HIV-positive patients after tuberculosis and cryptococcosis, and therefore has become an important acquired immune deficiency syndrome defining condition (Supparatpinyo et al., 1994). The clinical manifestation of *T. marneffei* infection is characterized by a variety of symptoms, including fever, anemia, weight loss, lymphadenopathy, hepatosplenomegaly, and skin lesions (Vanittanakom et al., 2006; Cooper and Vanittanakom, 2008). The disseminated infection caused by *T. marneffei* is incurable and usually fatal if untreated (Wong and Wong, 2011).

*T. marneffei* is capable of alternating between a filamentous and a yeast growth form, a process known as dimorphic transition. *T. marneffei* grows as multicellular mycelium showing mold colonies with soluble brick-red pigment at 25°C and switches to a unicellular yeast growth form showing colonies that are glabrous and beige-colored at 37°C (Wong et al., 1999). Yeast cells are found in infected patients, suggesting that they are the pathogenic form (Boyce and Andrianopoulos, 2013). However, conidia are considered to be the infectious agent, because they cause infection when inhaled into the host's lungs (Boyce and Andrianopoulos, 2013). Thus, the morphogenetic transition is critical for both pathogenicity and transmission of *T. marneffei* (Boyce and Andrianopoulos, 2015). In the laboratory, the dimorphism between the mycelia and yeast growth forms has previously been induced upon alteration of the culture temperature (Bugeja et al., 2013). Thus, *T. marneffei* is also categorized as thermally dimorphic fungi, along with *Histoplasma capsulatum, Blastomyces dermatitidis, Coccidioides immitis, Paracoccidioides brasiliensis*, and *Sporothrix schenckii* (Boyce and Andrianopoulos, 2015).

The dimorphic transition of *T. marneffei* is a complex process controlled by a suite of genetic elements (Andrianopoulos, 2002; Boyce and Andrianopoulos, 2013). The mechanism regulating the dimorphic transition between yeast and mycelial growth forms is not clearly understood. Moreover, little is known about the specific transcription factors regulating dimorphic transition in *T. marneffei*. In our previous study, we showed that *madsA* was involved in the phase transition of *T. marneffei* (Yang et al., 2014). The *madsA* gene belongs to the MADS-box gene family. MADS-box derived from the abbreviation of the first letter of four founding members of this family, i.e., *Mcm1, Agamous, Deficiens*, and *SRF* (Schwarz-Sommer et al., 1990). Members of in this gene family contain a conserved sequence that encodes a DNA-binding domain typically containing 56–60 amino acids (Shore and Sharrocks, 1995). Some genes belonging to this family have been reported in fungi, such as *FgMCM1*, a key regulator of conidiation and virulence of *Fusarium graminearum* (Yang et al., 2015). The *Aspergillus fumigatus rlmA* gene is required for regulation of the cell wall integrity (Rocha et al., 2016). In our previous study mentioned above, we found that the expression level of *madsA* was significantly high both in yeast growth phase and during yeast-to-mycelium transition (Yang et al., 2014). In addition, overexpression of *madsA* was able to induce mycelial growth at 37°C, at which temperature the wild-type (WT) strain grew as yeast cells (Yang et al., 2014). However, the roles of *madsA* in dimorphic transition and the underlying mechanism remain to be clearly elucidated.

In this study, we constructed the deletion mutant and the complemented mutant of *madsA* in *T. marneffei* to determine its function in morphogenesis, dimorphic transition, and stress response. We found that the deletion mutant exhibited a faster transition from yeast (37°C) to mycelium (25°C) and had abnormal morphogenesis. The latter was characterized by diminished conidiation, slower conidial germination, and stronger tolerance of conidia to oxidative stress. We also compared the transcriptomic profiles between the *madsA* deletion mutant and the WT strain during different processes. The large dataset obtained from transcriptomic profile analysis provided rich information and revealed that the deletion of *madsA* affected genes involved in transmembrane transport, oxidation-reduction process, protein catabolic process and response to oxidative stress. Taken together, our data show that *madsA* function as a regulator of yeast-to-mycelium transition and is closely related to conidiation and germination in *T. marneffei*.

## Materials and methods

### Strains, media, and culture conditions

The *T. marneffei* strain PM1 was isolated from an HIV-negative patient suffering from culture-documented penicilliosis in Hong Kong (Woo et al., 2010). The genome of PM1 has been assembled by combining Sanger, Illumina, and PacBio sequencing technologies in our previous studies (Woo et al., 2011; Yang et al., 2014). Here, we used PM1 as a WT strain and a parental strain to construct mutant strains. All strains were maintained at 25°C on Sabouraud Dextrose Agar (SDA) (Becton, Dickinson and Company, USA), which was also used for preparing conidial inocula (see below). Sabouraud Dextrose Broth (SDB) (Becton, Dickinson and Company, USA) was employed for experiments involving liquid cultures of WT. For long-term storage, mycelia of a given strain were suspended in 30% (w/v) sterile glycerol and frozen at −80°C.

### Construction of *MadsA* deletion mutant

**Figure 1:**
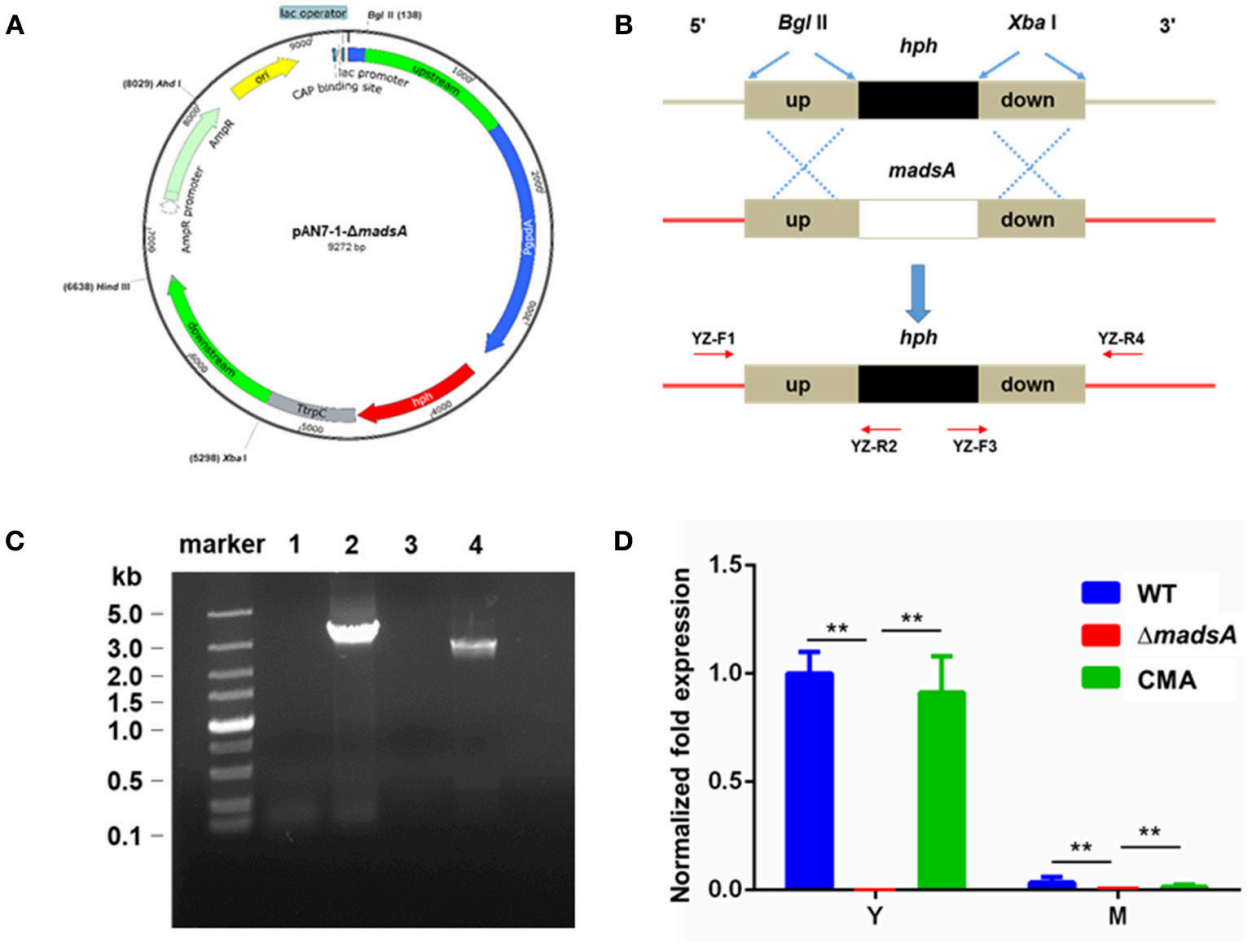
Deletion of *madsA* gene in *T. marneffei* by homologous recombination. **(A)** Structure of recombinant plasmid pAN7-1-Δ*madsA*. **(B)** The process of homologous recombination. **(C)** Detection of the recombinant fragments in WT and Δ*madsA* strains. Lane 1 and Lane 2: suspected PCR products from *madsA* upstream to *hph* in WT and Δ*madsA*, respectively. Lane 3 and Lane 4: suspected PCR products from *hph* to *madsA* downstream in WT and Δ*madsA*, respectively. **(D)** Expression levels of *madsA* in WT, Δ*madsA* and CMA strains. **p < 0.0001. Error bar represents standard deviation (SD) from three biological replicates. CMA, complemented strain; *hph*, hygromycin B phosphotransferase; M, mycelium; PCR, polymerase chain reaction; WT, wild-type; Y, yeast.

The *madsA* deletion mutant was constructed by the homologous recombination approach (Woo et al., 2006; Lau et al., 2013). Firstly, we used pAN7-1 plasmid as the vector for construction a recombinant plasmid harboring a hygromycin B phosphotransferase gene (*hph*) with *madsA* flanking sequences as adjacent sequences at each of its ends. The expression of the *hph* gene in pAN7-1 vector was controlled by *Aspergillus nidulans gpd* and *trpC* expression signals (Punt et al., 1987; Lau et al., 2013). The Integrated Genome Browser (IGB, http://bioviz.org/igb/) software was utilized to search the *madsA* open reading frame and its flanking sequences from the PM1 genome (AGCC00000000) for primer design (Woo et al., 2011; Yang et al., 2014). The *madsA* upstream (1,240 bp) and downstream (1,276 bp) flanking sequences were amplified from WT genomic DNA by polymerase chain reactions (PCRs) using the primer pairs of KO-F1/KO-R1 (each containing a *Bgl*II cleavage site) and KO-F2/KO-R2 (each containing a *Xba*I cleavage site) (see underlined sequences Table 1), respectively. The PCR was performed using PrimeSTAR Max DNA Polymerase (Takara, Japan) in accordance with the manufacturer's instruction. PCR protocol was composed of an initial cycle at 98°C for 10 s; 30 cycles at 98°C for 10 s, 58°C for 5 s, and 72°C for 10 s; 72°C for 5 min; and hold at 4°C. The pAN7-1 vector was linearized by *Bgl*II and *Xba*I digestion, respectively. Ligation of the linearized pAN7-1, *madsA* upstream and downstream flanking sequences was accomplished using ClonExpressTM II One Step Cloning Kit (Vazyme, China). The resulting plasmid, named pAN7-1-Δ*madsA* (Figure 1A), contained the *hph* gene and the two homologous fragments to *madsA* flanking sequences, and was used for transformation of WT protoplasts to knock out *madsA*.

**Table 1:** List of primers used in this study.

**Primer name**	**Sequence (5′to 3′)**	**Purpose**
**FOR PCR EXPERIMENT**		
KO-F1	tgaagt*aatctctgcAGATCT*GAATCCAGCACAGAGCTTATGCA^a^	pAN7-1 cloning
KO-R1	tatttcagtgtcgaaAGATCTCGTGTACAAGGGTATCTTAGTGTGTAAT^a^	pAN7-1 cloning
KO-F2	gaggtaatccttcttTCTAGATATTTCCGATACGTTGTGACTCGG^b^	pAN7-1 cloning
KO-R2	cagtacacgaggactTCTAGATCCATGTTCCACAGCTGGCC^b^	pAN7-1 cloning
YZ-F1	GTTTGCTGTGCTGTGCCACGATT	Deletion transformant confirmation
YZ-R2	AAGCTGCCTACCAGGGACTGAGGG	Deletion transformant confirmation
YZ-F3	ATTTCGGCTCCAACAATGTCCTG	Deletion transformant confirmation
YZ-R4	CGGCAACCCAAGTAGTTCCCACC	Deletion transformant confirmation
CM-F	gaggtaatccttcttTCTAGAATACATCATTGCTTCCCCTCTACC^c^	pAN8-1 cloning
CM-R	gtactgagagtgcacCATATGATACCCAAGCGATATGACAAACAC^d^	pAN8-1 cloning
**FOR qPCR EXPERIMENT**		
*act*-F	CCCAAGTCCAACAGAGAGAAG	Endogenous reference
*act*-R	GGAAGCGTACAAGGACAAGA	Endogenous reference
*madsA*-F	CCGCGAAAGGACAAAGTATCT	Detect the expression level
*madsA*-R	CTCCAGAGCATGGACGAATTT	Detect the expression level
*hph*-F (primer 1)	CCGCAAGGAATCGGTCAATA	Determinate the *hph* copy number
*hph*-R (primer 1)	GGTGTCGTCCATCACAGTTT	Determinate the *hph* copy number
*hph*-F (primer 2)	GCTTTCAGCTTCGATGTAGGA	Determinate the *hph* copy number
*hph*-R (primer 2)	CGATGCAAAGTGCCGATAAAC	Determinate the *hph* copy number
*hph*-F (primer 3)	TGTCGGGCGTACACAAATC	Determinate the *hph* copy number
*hph*-R (primer 3)	GGCGTCGGTTTCCACTATC	Determinate the *hph* copy number
PMG0022150^#^-F	GCCATCTCCGTCCTATCTATCA	Validation of RNA-seq data
PMG0022150^#^-R	GAATCAGGACCAACGGAAGTAG	Validation of RNA-seq data
PMG0030080^#^-F	GGGAATCTGGGAAAGGTAGAAG	Validation of RNA-seq data
PMG0030080^#^-R	TGTAGGACGGAATGTAGGTAGT	Validation of RNA-seq data
PMG0040630^#^-F	CGACCCAAACACCAACTTTAC	Validation of RNA-seq data
PMG0040630^#^-R	CCAGGAGGGAAACTGAACTT	Validation of RNA-seq data
PMG0062960^#^-F	GCCTCGTCGGCATTATCTT	Validation of RNA-seq data
PMG0062960^#^-R	GACGCGTAAGTCCTCAAAGT	Validation of RNA-seq data
PMG0080490^#^-F	CAAATGTGACGGCTACCTACTC	Validation of RNA-seq data
PMG0080490^#^-R	GTGAAAGTCTGAAGTCCGAAGAG	Validation of RNA-seq data
PMG0131830^#^-F	CTCGTGGCTTCTCCGTTAAA	Validation of RNA-seq data
PMG0131830^#^-R	GATGGATCACGCAGGAAGAA	Validation of RNA-seq data
PMG0180910^#^-F	GTTACTGGAGAAACACCGAAGG	Validation of RNA-seq data
PMG0180910^#^-R	GATGTTGCTGGACGGTCTTATT	Validation of RNA-seq data
PMG0430050^#^-F	CAGAGAAGTGGCAAGGTATTGA	Validation of RNA-seq data
PMG0430050^#^-R	CTGATCAGCACAGAGAGGATTAC	Validation of RNA-seq data
PMG0600380^#^-F	TGACACGATCCGTGCAAATA	Validation of RNA-seq data
PMG0600380^#^-R	TGATGCTTGGTGGTAGTGAAG	Validation of RNA-seq data

a *Lowercase letters: the sequences are homologous to pAN7-1. Underlined sequences: cleavage sites of BglII*.

b *Lowercase letters: the sequences are homologous to pAN7-1. Underlined sequences: cleavage sites of XbaI*.

c *Lowercase letters: the sequence is homologous to pAN8-1. Underlined sequences: cleavage sites of XbaI*.

d *Lowercase letters: the sequence is homologous to pAN8-1. Underlined sequences: cleavage sites of NdeI*.

# *The gene ID in wild-type genome (AGCC00000000) (Woo et al., 2011; Yang et al., 2013, 2014)*.

*F, forward primers; hph, hygromycin B phosphotransferase gene; PCR, polymerase chain reaction; R: reverse primers*.

The WT strain was transformed with the plasmid pAN7-1-Δ*madsA* based on the protocol previously described (Borneman et al., 2001) with several modifications. Briefly, ~1 × 10^7^ PM1 conidia harvested from SDA were inoculated into 100 ml SDB and cultured for 18–20 h at 37°C and 150 rpm. The optimal time for protoplast preparation was when most of the germlings had short primary germ tubes. Highly branched germlings could be removed by filtration through Miracloth (Calbiochem, Germany). The culture was centrifuged and washed twice with 0.6 M MgSO_4_. The lysing enzyme mixture included lytic enzyme (Sigma, USA) and bovine serum albumin (9 dingchem, China) at final concentrations of 5 mg/ml and 1.2 mg/ml, respectively, in osmotic buffer (1.2 M MgSO_4_ and 10 mM sodium phosphate, pH 5.8). The 15 ml of lysing enzyme mixture with the germlings grown in 100 ml of SDB medium were incubated for 2 h at 28°C with gentle agitation. Protoplasts were harvested, washed and resuspended in STC buffer (1.2 M Sorbitol, 10 mM CaCl_2_ and 10 mM Tris-HCl pH 7.5) in a concentration of 1 × 10^7^−1 × 10^8^/ml. The 100 μl of protoplast suspension was incubated on ice with 5–8 μg (in 15 μl) of linearized pAN7-1-Δ*madsA* for 50 min. Then 1.25 ml of PEG/CaCl_2_ solution (60% PEG 4000, 10 mM CaCl_2_ and 10 mM Tris-HCl pH 7.5) was added and incubated at room temperature for 30 min. Subsequently, the protoplasts were diluted with 5 ml of STC buffer and plated onto SDA supplemented with 200 μg/ml of hygromycin B and 0.6 M sorbitol. The plates were incubated at 37°C for 10 d. The hygromycin-resistant transformants were consecutively cultured on the SDA plates without and with hygromycin for 5 and 1 passages, respectively. The resulting stable-resistant colonies were selected, subjected to confirmation tests by PCR and sequencing with the primers YZ-F1/R2 and YZ-F3/R4 (Table 1, Figure 1B). To ensure a complete absence of *madsA* mRNA, quantitative reverse transcription PCR (RT-qPCR) was subsequently performed with triplicate in each of the three independent experiments. The *hph* copy number in the deletion mutant was verified by a qPCR (Stefano et al., 2016). Three pairs of primers (Table 1) were designed for *hph* gene to avoid the influence of amplification efficiency. The single copied β-actin (*act*) gene was used as an endogenous control (Kummasook et al., 2011; Yang et al., 2014). The confirmed *madsA* deletion strain was named as Δ*madsA*.

### Construction of *MadsA* complemented mutants from Δ*madsA* mutant

For genetic complementation of the deletion mutant, plasmid pAN8-1 carrying bleomycin-resistant gene (*ble*) was used to construct the pCM-*madsA* vector (Jain et al., 1992; Lau et al., 2013). The CM-F and CM-R primers (Table 1) containing *Xba*I and *Nde*I site, respectively, were used to amplify the *madsA* gene consisting of the promotor and 3′ region from WT genomic DNA. The PCR product (982 bp) was inserted into pAN8-1. The plasmid pCM-*madsA* linearized with *Ahd*I was transformed into the Δ*madsA* mutant using protoplast transformation as described in the previous section. After transformation, the transformants were recovered in SDB containing 0.6 M sorbitol and incubated at 25°C, 150 rpm for 1–2 days (Nimmanee et al., 2015). Regenerated protoplasts were plated on SDA containing 100 μg/ml of bleomycin (Invitrogen, USA) and were cultured for 7–10 days. Strains were isolated from the colonies with bleomycin resistance through single spore separating. After the isolation, colonies similar to WT strain were selected and confirmed by PCR and sequencing. The expression of *madsA* in the complemented strains was further confirmed by RT-qPCR. One confirmed *madsA*-complemented strain was named as CMA and used for the study.

### Phase transition treatments

**Figure 2:**
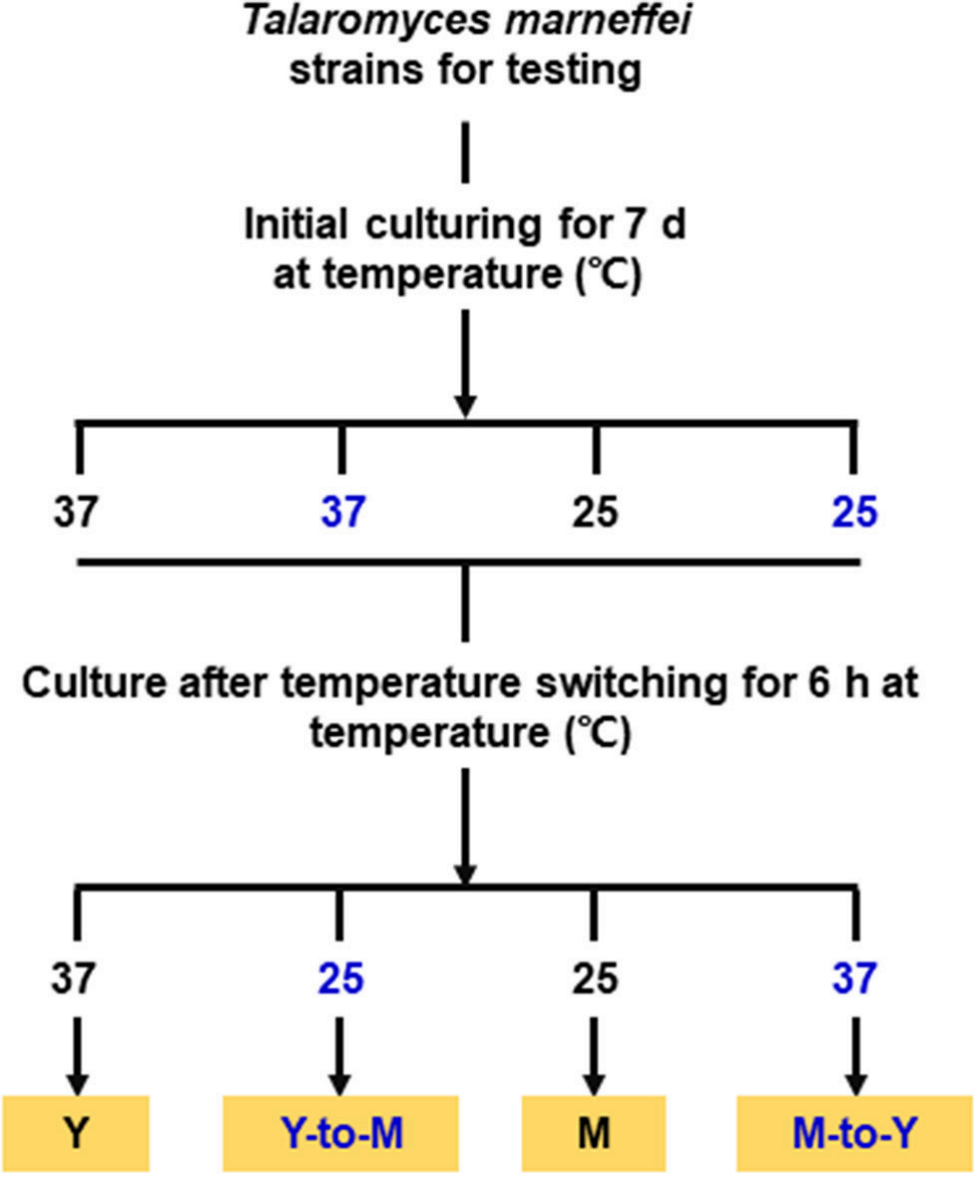
Flow chart for the phase transition treatments. M, mycelium; Y, yeast.

The WT, Δ*madsA* mutant and *madsA* complemented CMA strains were subjected to phase transition treatments by temperature switching culture for 6 h according to our previous study (Yang et al., 2014). Each strain was initially cultured on the SDA plates at 37°C (or 25°C) for 7 d in order to get the stable yeasts (or mycelial organisms). For temperature switching experiments, yeasts grown at 37°C were transferred to 25°C for 6 h (yeast-to-mycelium, Y-to-M), and mycelia grown at 25°C were transferred to 37°C for 6 h (mycelium-to-yeast, M-to-Y). As the control group, the stable yeasts (or mycelial organisms) were subsequently cultured at the initial temperatures for 6 h as well. The cultures were grown in continuity at 37°C as yeasts (yeast, Y) or grown in continuity at 25°C as mycelia (mycelium, M). A simplified flow chart was shown in the Figure 2. Three independent experiments were performed.

### Colony growth and conidial germination

The conidial suspension of WT, Δ*madsA* and CMA strains was prepared as follows. The surfaces of colonies cultured on SDA for 7 d at 25°C were scraped with a cotton swab to collect conidia (Suwunnakorn et al., 2015). The swabs were immersed in 10 mM phosphate buffered saline (PBS) to obtain conidial suspension, which then was filtered through sterile miracloth to remove mycelial fragments. Conidia of each strain were counted using a hemocytometer chamber and diluted to the same concentrations. For quantification of colony growth, 5 μl of 1 × 10^4^ conidia/ml of WT, Δ*madsA* and CMA strains were dropped on SDA then incubated at either 25 or 37°C. The colony diameters were measured on 2–7 d of inoculation with three biological replicates (Suwunnakorn et al., 2014).

A modified germination assay was performed based upon a previously described method (Boyce and Andrianopoulos, 2007). Briefly, ~1 × 10^6^ conidia were inoculated into 4 ml of SDB then incubated at either 25 or 37°C. After 8 h, the numbers of germinating conidia were counted with 4-h interval in a population of 100 by using microscope (Revolve FL, Echo-Labs, USA) to measure the rate of germination. Three independent experiments were performed.

### Susceptibility to oxidative stress and osmotic stress

The assay for tolerance of the conidia to oxidative stress was performed based on the method previously described. (Woo et al., 2010). Conidial suspensions of WT, Δ*madsA* and CMA strains were adjusted to 1 × 10^6^ conidia/ml in 10 mM PBS (pH 7.0) containing 25 mM hydrogen peroxide (H_2_O_2_). A 100 μl aliquot from each treatment was taken at 5-min interval, diluted to 100-fold (about 1 × 10^4^ conidia/ml) in 10 mM PBS. 100 μl of the suspension was plated onto SDA plates, then cultured at 37°C for 7 d. The visible colonies on the plates were counted. The relative survival of conidia capable of forming visible colonies was calculated. Three independent experiments were performed.

To test the chronic effects of H_2_O_2_ treatment on mycelia and yeast cell growth, 5 μl of conidial suspension (1 × 10^5^−1 × 10^2^ conidia/ml) of WT, Δ*madsA* and CMA strains was spotted on the surface of the SDA plate supplemented with 1.25, 2.5, 5, 10, or 20 mM H_2_O_2_, respectively. Then, the conidia were cultured on SDA plates for 7 d at 25°C or 37°C (Pongpom et al., 2013).

Under the similar experimental conditions, the susceptibility to osmotic stress was tested. Five microliters of conidial suspension (1 × 10^5^−1 × 10^2^ conidia/ml) of each strains was spotted on the surface of the SDA plate supplemented with 0.125, 0.25, 0.5, 1, or 2 M sorbitol, respectively. Then, the conidia were cultured on SDA plates for 7 d at 25 or 37°C.

### RNA preparation

Total RNA was isolated under the four conditions described above in phase transition treatments, including Y, M, Y-to-M, and M-to-Y, respectively. The total RNAs were extracted from each condition for three independent biological replicates using the Fungal RNA Kit (Omega Bio-Tek, USA) and following the manufacturer's instructions for DNase I digestion to eliminate genomic DNA contamination.

### Transcriptome sequencing

For sequencing, the total RNA was extracted from WT and Δ*madsA* strains cultured under four temperature treatment conditions, respectively. Three biological replicates were performed for each trial, from which the independent library was constructed for sequencing and analysis, respectively. In total, we made 24 cDNA libraries. The RNA integrity numbers (RIN) of all samples were analyzed using a 2100 Bioanalyzer (Agilent Technologies, USA). The cDNA library construction (Vazyme kit) and sequencing (Illumina kit) were performed by Mega Genomics (Beijing, China). The libraries were sequenced on an Illumina HiSeq 4000 platform using a strand-specific paired-end sequencing strategy.

### Transcriptome analysis

The raw reads were assessed for quality by FASTQC v0.11.5 (http://www.bioinformatics.babraham.ac.uk/projects/fastqc/) and filtered to remove low-quality reads with Trimmomatic v0.36 (Bolger et al., 2014). Filtered reads were splice-aware aligned to the reference genome of *T. marneffei* (Yang et al., 2014) by using STAR v2.5.3a (Dobin et al., 2013). The gene expression levels were calculated by using FeatureCounts v1.5.2 (Liao et al., 2014) and then normalized based on the FPKM (fragments per kilobase of transcript per million fragments mapped) method (Trapnell et al., 2010). Differentially expressed genes were detected by DESeq2 (Love et al., 2014) in Bioconductor packages. Genes were considered to be differentially expressed between Δ*madsA* strain and WT strain if the Benjamini–Hochberg adjusted *p*-value was below 0.05 and the fold change was above 2. Gene ontology (GO) term enrichment analysis on differentially expressed genes was performed with GOseq (Young et al., 2010; Yang et al., 2013), also available from Bioconductor packages using a background set annotated by running InterProScan v5.27-66.0 (Zdobnov and Apweiler, 2001). The enriched GO terms were summarized and visualized using REVIGO (Supek et al., 2011). Raw sequence data were deposited in the NCBI Sequence Read Archive database (SRA) under the accession number of SRP131610.

### RT-qPCR

For each strain under all phase switching conditions, the cDNA was synthesized with random primer by reverse transcription of total RNA (1 μg) using Prime Script^®;^ RT reagent kit (TaKaRa, Japan). The cDNA (80 ng) samples were amplified in reaction mixtures containing SYBR Premix Ex Taq II (Takara, Japan) using an ABI Step One Real Time PCR System. The reaction was carried out with one cycle at 95°C for 15 s, 40 cycles at 95°C for 5 s and then 60°C for 30 s. A non-cDNA synthesis control was included to ensure that no DNA contamination existed. Primer sequences used for amplification of specific genes were shown in Table 1. The *act* gene was used as an endogenous control for gene expression analysis using the 2^(−ΔΔ*CT*)^ analysis method as suggested by a previous study (Livak and Schmittgen, 2001; Dankai et al., 2015). Triplicate in each test and three independent experiments were carried out.

### Statistical analyses

Statistical analyses were performed using IBM SPSS Statistics 20.0 software program (IBM, USA). The Student's *t*-test or Chi-square test was used to analyze the appropriate data. A *p* < 0.05 was considered statistically significant.

## Results

### The *MadsA* gene deletion mutant and complemented strain

The *madsA* gene consists of two exons and one intron. It encodes a protein of 109 amino acids. The protein sequence was deposited in the GenBank database with an accession number of KFX52038.1 (Yang et al., 2014). The structure of *madsA* gene is showed in Figure S1. To study the role of *madsA* in *T. marneffei*, we constructed the deletion mutant by replacing the coding sequence of *madsA* with *hph* cassette (Figure 1B). The putative transformants were verified by PCR amplification using internal and external primers specific to *madsA* and *hph* genes (Figure 1B). In Δ*madsA* mutant, the *hph* gene was found to be integrated within the genome by homologous recombination (Figure 1C). The copy number of *hph* gene inserted in the mutant was identical to the *act* (Table S1). *MadsA* expression levels in Δ*madsA* and CMA strains were compared against the WT strain by RT-qPCR, respectively. The *madsA* transcript was undetectable in Δ*madsA* mutant, which was significantly different from those of the WT and CMA strains (Figure 1D). These results verified that the *madsA* gene deletion was successfully deleted and a single copy of *hph* gene was inserted into Δ*madsA* mutant. Furthermore, the complemented strains that showed colonies similar to the WT were screened by PCR for the *madsA* and *ble* integration (data not shown). The expression level of *madsA* in CMA strain was close to WT strain under yeast and mycelial culture conditions (Figure 1D). The Δ*madsA* and CMA strains were used in further studies.

### Phenotypic characterization of Δ*madsA* mutant in dimorphic transition

**Figure 3:**
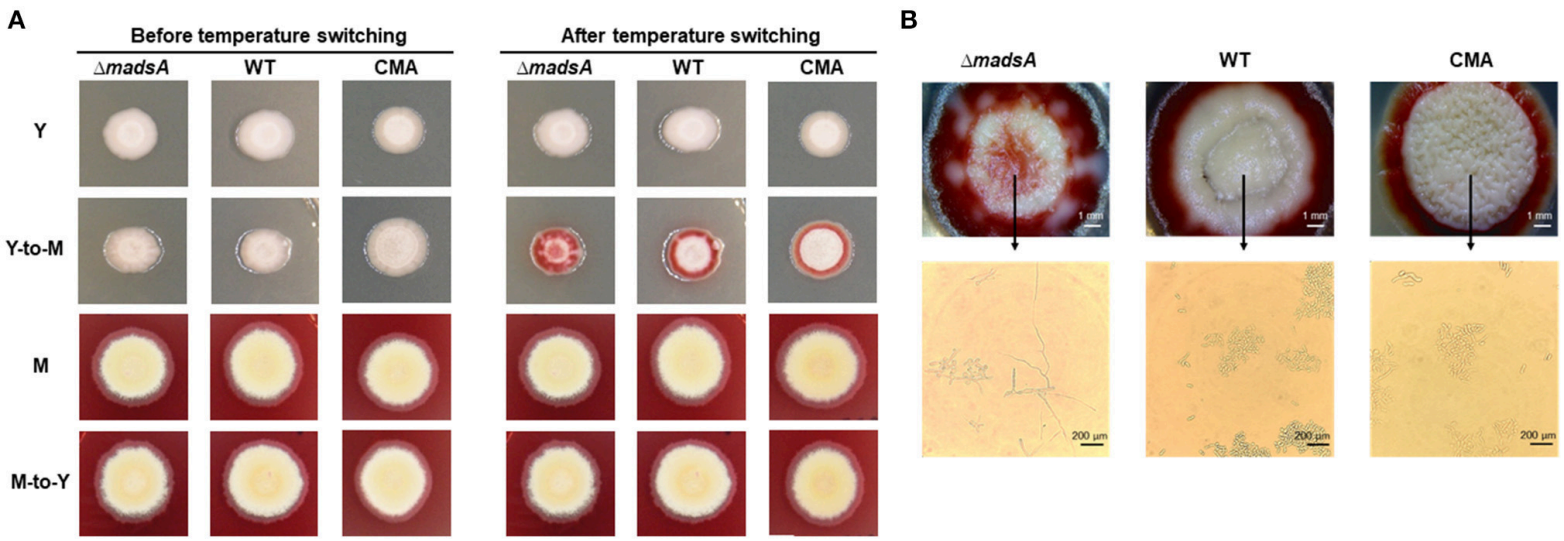
Morphological phenotypes of the Δ*madsA*, WT and CMA strains in phase transition treatments. **(A)** The colonial morphologies of the three strains in four conditions. **(B)** The colonial and microscopic morphologies of the three strains in Y-to-M transition treatment. CMA, complemented strain; M, mycelium; WT, wild-type; Y, yeast.

To assess the roles of *madsA* in dimorphic morphogenesis of *T. marneffei*, we compared the morphologies among the Δ*madsA* mutant, WT and CMA strains under four different culture conditions as illustrated in Figure 2. Except in the Y-to-M transition, we did not find any significant morphological differences among the three strains at the macroscopic or microscopic level. The morphologies of the complemented strain CMA were similar to the WT strain. Specifically, we found that all strains exhibited glabrous and beige-colored colonies without pigments (Figure 3A) at yeast growth. Microscopic examination of Δ*madsA* cultures showed that the yeast-like cells were the same as those of other strains (data not shown). Similarly, regarding the stable mycelial culture condition, the Δ*madsA* strain formed mycelium-like colony secreting diffusible red pigment (Figure 3A) that was similar to WT and CMA strains. The microscopic morphology of Δ*madsA* strain was indistinguishable from the controls either (data not shown). However, during the Y-to-M transition, Δ*madsA* mutant showed significant differences compared with the WT and CMA strains. After the temperature switch disposition, almost the entire colony of the Δ*madsA* strain turned red, whereas the WT and CMA colonies only displayed marginal reddening (Figure 3A). The detailed morphology was further compared under microscopy. Mycelia was observed in Δ*madsA* strain, indicating that the conversion from yeast to mycelial phase had occurred; cells of the WT and CMA strains maintained as yeasts under the same condition (Figure 3B). By contrast, in the reciprocal experiment from M-to-Y, Δ*madsA* strain exhibited a phenotypic characteristic nearly identical to the other strains (Figure 3A). Three independent experiments were performed, and the phenotypic characteristics were reproducible. In general, the absence of *madsA* led to a faster Y-to-M transition, but had no detectable impact on phenotype during the M-to-Y transition.

### *madsA* deletion leads to a delay in conidia production and conidial germination

**Figure 4:**
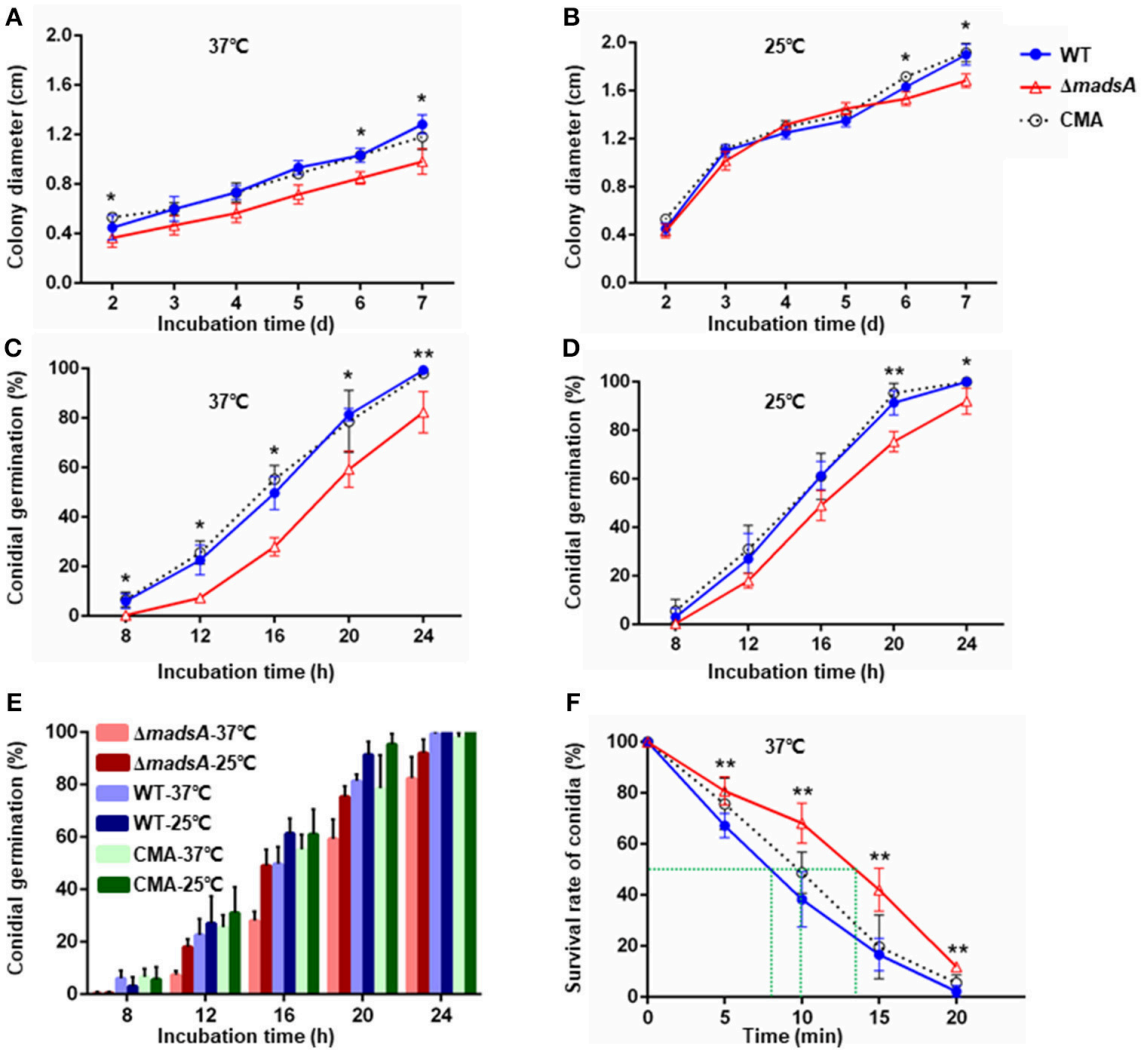
Characteristic of Δ*madsA*, WT and CMA strains in growth, conidial germination and conidial susceptibility to H_2_O_2_. **(A,B)** Radial growth (colony diameter) at 37 and 25°C. **(C,D)** Conidial germination at 37 and 25°C. **(E)** Comparison of the germination rates among three strains at different culture temperatures. **(F)** Survival rates of conidia after H_2_O_2_ treatment for different times. *p < 0.05. **p < 0.0001. Error bar represents SD from three biological replicates. CMA, complemented strain; M, mycelium; SD, standard deviation; WT, wild-type; Y, yeast.

To investigate the role of *madsA* gene in growth, we compared the colony diameters of Δ*madsA*, WT and CMA strains cultured at 37 and 25°C for 2–7 d, respectively. At 37°C, there was a reduction in colony diameter of the Δ*madsA* mutant in comparison with the WT and CMA strains at different incubation time points (Figure 4A).

**Figure 5:**
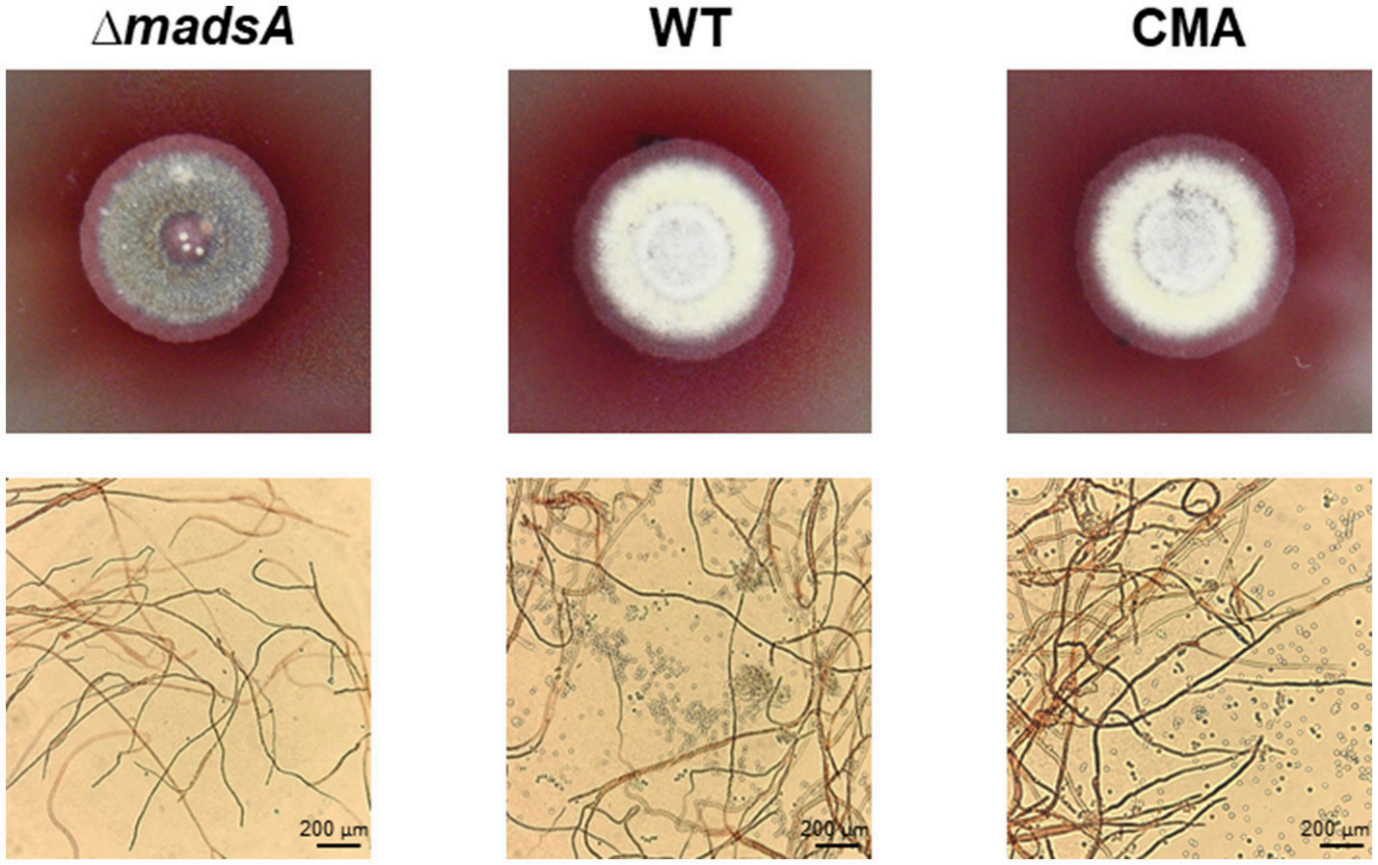
Phenotype of Δ*madsA*, WT and CMA strains after 4 d growth at 25°C. The Δ*madsA* strain showed a relatively smooth colony with reduced conidial production, whereas WT and CMA showed visibly rough colonies with abundant conidia. CMA, complemented strain; M, mycelium; WT, wild-type; Y, yeast.

At 25°C, the colonies of Δ*madsA* mutant had a similar diameter to other strains (Figure 4B). Although the diameters of WT and Δ*madsA* strains were statistically different at the endpoint of incubation period, the growth curves showed similar kinetics. It is worth noting that the Δ*madsA* mutant showed relatively smooth colony morphology whereas the WT and CMA strains showed rough colony morphology after 4 d inoculation at 25°C. In addition, the colonies of Δ*madsA* mutant were gray with loss of the yellow color on the colony surface (Figure 5). A reduction in conidial production of Δ*madsA* mutant was found by microscopic observation (Figure 5). Despite the initial reduction in conidia, after 7 d all strains were producing conidia. The Δ*madsA* strain was not substantially different from the strains containing the *madsA* gene in colony morphology during subsequent culture at 25°C (Figure 3A).

To determine whether the *madsA* affected conidial germination, the rates of germination were assessed. The germination rates of WT and CMA strains were not significantly different (*p* > 0.05). However, the Δ*madsA* strain showed delays in germination at both 37°C (Figure 4C) and 25°C (Figure 4D). In particular, significant differences (*p* < 0.05) were found at all sampling time points at 37°C. When comparing the germination rates between the WT and Δ*madsA* strains at different culture temperatures (Figure 4E), we found that, on average, the Δ*madsA* took about 4 h longer than WT to achieve the same germination rates at 37°C. As mentioned above, the colonies of Δ*madsA* mutant were slightly smaller than that of WT. This might be due to the delay in germination of Δ*madsA* mutant. To test the hypothesis, the synchronizing cultures were applied at 37°C. The conidia of Δ*madsA* strain had a 4 h incubation in SDB prior to cultured on SDA plates. As a result, the colony diameter of Δ*madsA* strain was similar to that of WT (Figure S2).

### The role of *madsA* in susceptibility to oxidative stress and osmotic stress

The survival rates of conidia at different incubation times in H_2_O_2_ were plotted (Figure 4F). The treatment time resulting in a 50% reduction in conidial survival were about 8 min for WT, 10 min for CMA strain and about 13.5 min for the Δ*madsA* mutant, respectively. Thus, the Δ*madsA* conidia displayed a stronger resistance to H_2_O_2_ than the WT strain. The chronic treatment of H_2_O_2_ revealed that neither Δ*madsA* nor WT strains could grow at H_2_O_2_ concentration ≥2.5 mM regardless of the culture temperature. The colony morphology of Δ*madsA* and WT strains had no obvious difference in the presence of 2 mM H_2_O_2_ (data not shown).

To determine the function of *madsA* in response to osmotic stress, the strains were grown on media supplemented with gradient concentration of sorbitol at 25 or 37°C, respectively. The colony morphology and pigmentation of Δ*madsA* mutant grown under osmotic stress was similar to the WT (Figure S3). The microscopic visualization showed no significant difference either. The CMA strain exhibited a phenotype identical to the WT (data not shown). Thus, the susceptibility of the Δ*madsA* mutant to osmotic stress was almost the same to that of WT.

### Transcriptional profiling of the *MadsA* deletion mutant

To gain an insight into to the molecular processes regulated by *madsA* underlying the observed phenotypic characteristics, RNA sequencing (RNA-seq) was performed on total RNA extracted from the WT and Δ*madsA* strains under the four experimental conditions as mentioned in the Figure 2. Table S2 showed the quantity and quality of total RNA for each sample. The RIN values for most samples were over 8.0 (22/24). The mean RIN value of the three replicates under each treatment condition was larger than 8.0, respectively, suggesting the RNA had good quality for transcriptome analysis.

**Figure 6:**
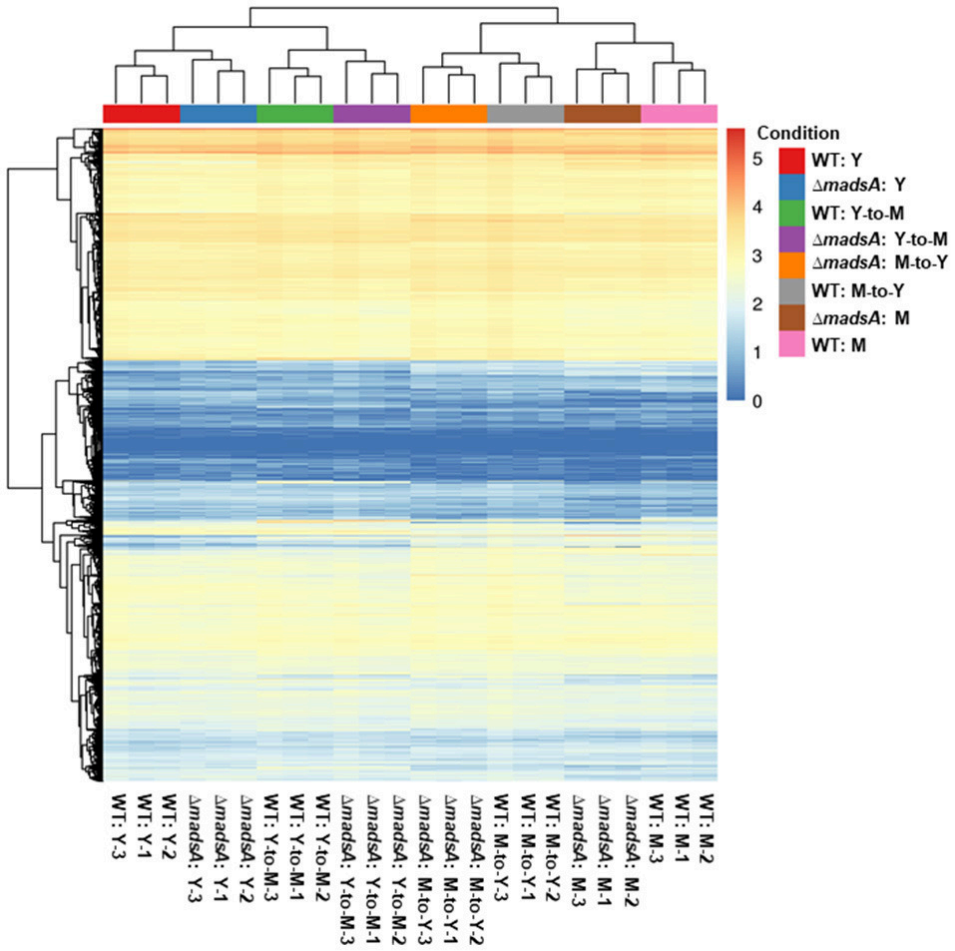
The gene expression levels of Δ*madsA* and WT strains under four conditions. The correlation of gene expression patterns and levels between biologically repeated samples was consistent. The 0 (minimum) to 5 (maximum) bar represents relative abundance of gene expression. M, mycelium; WT, wild-type; Y, yeast.

For each sample, we obtained 11.06–18.20 million pairs of raw reads. After removing the low quality reads and adapter sequences, between 9.66 and 15.05 million pairs of clean reads were left for each sample (Table S3). Quality control parameters indicated that the resulting gene transcript data were reliable. In addition, there was good correlation of gene expression patterns and levels between biologically repeated samples, and the heat cluster map indicated high repeatability of the sequencing (Figure 6).

Prior to identifying the differentially expressed genes (DEGs) between Δ*madsA* and WT, gene expression levels in different samples were calculated using FPKM (Trapnell et al., 2010). The number of genes expressed in each sample was more than 8,200, which represented most of the genes predicted from the sequence analysis of the *T. marneffei* genome (Yang et al., 2014). Expression levels of these genes under different treatments were compared using the DESeq2 (Love et al., 2014).

**Figure 7:**
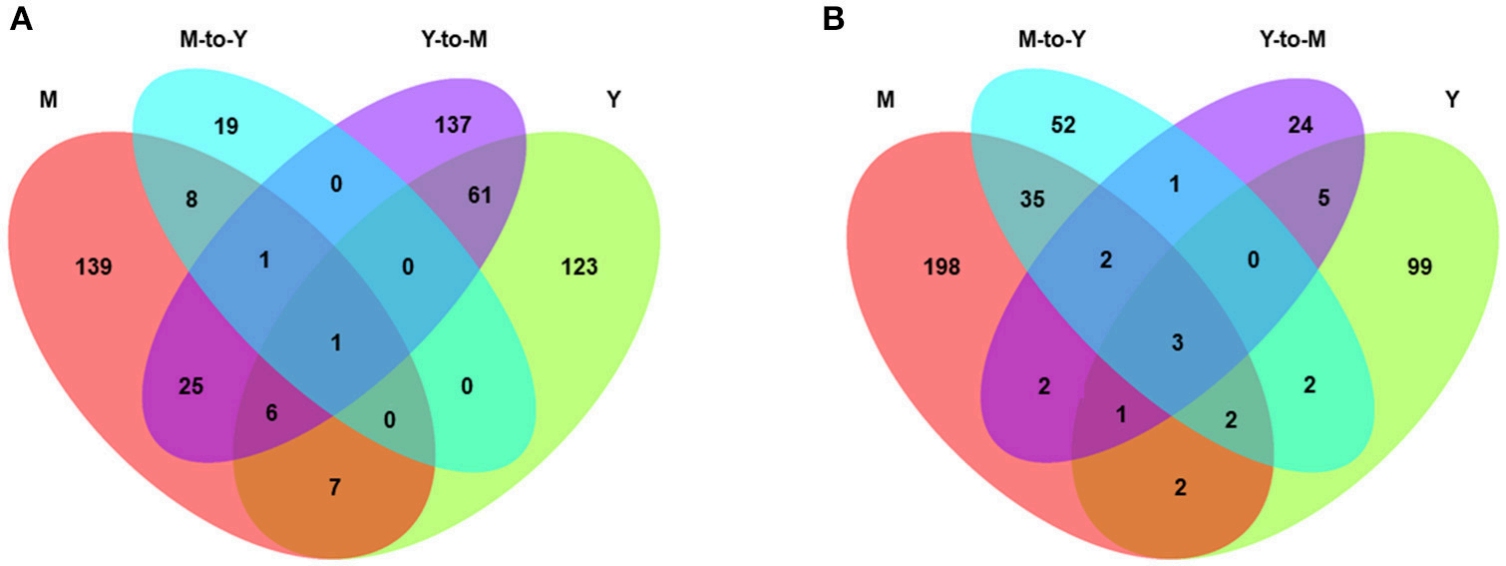
Transcriptome profiles of differentially expressed genes (DEGs) of Δ*madsA* in comparison with WT under four conditions. **(A)** The unique and common upregulated DEGs under four conditions. **(B)** The unique and common downregulated DEGs under four conditions. M, mycelium; Y, yeast.

We identified a total of 869 genes that were differentially expressed between WT and Δ*madsA* strains. Compared with the WT strain, 198 genes of Δ*madsA* were upregulated and 114 were downregulated in yeast growth. Likewise, in Y-to-M growth, 231 genes were upregulated and 38 were downregulated. However, many fewer DEGs were identified in M-to-Y transition, of which 29 genes were upregulated and 97 were downregulated. As for mycelial growth, 187 genes of Δ*madsA* were upregulated and 245 were downregulated. A Venn diagram (Figure 7) shows both unique and common DEGs in different comparisons at Y, Y-to-M, M, and M-to-Y.

To analyze the function of DEGs showing up or down regulation in response to *madsA* knocked out, we performed GO enrichment analysis. Individual GO terms were considered to be enriched if their corrected *p*-value was < 0.05, Tables S4–S7 contains a list of the enriched GO terms under Y, Y-to-M, M, and M-to-Y, respectively.

**Figure 8:**
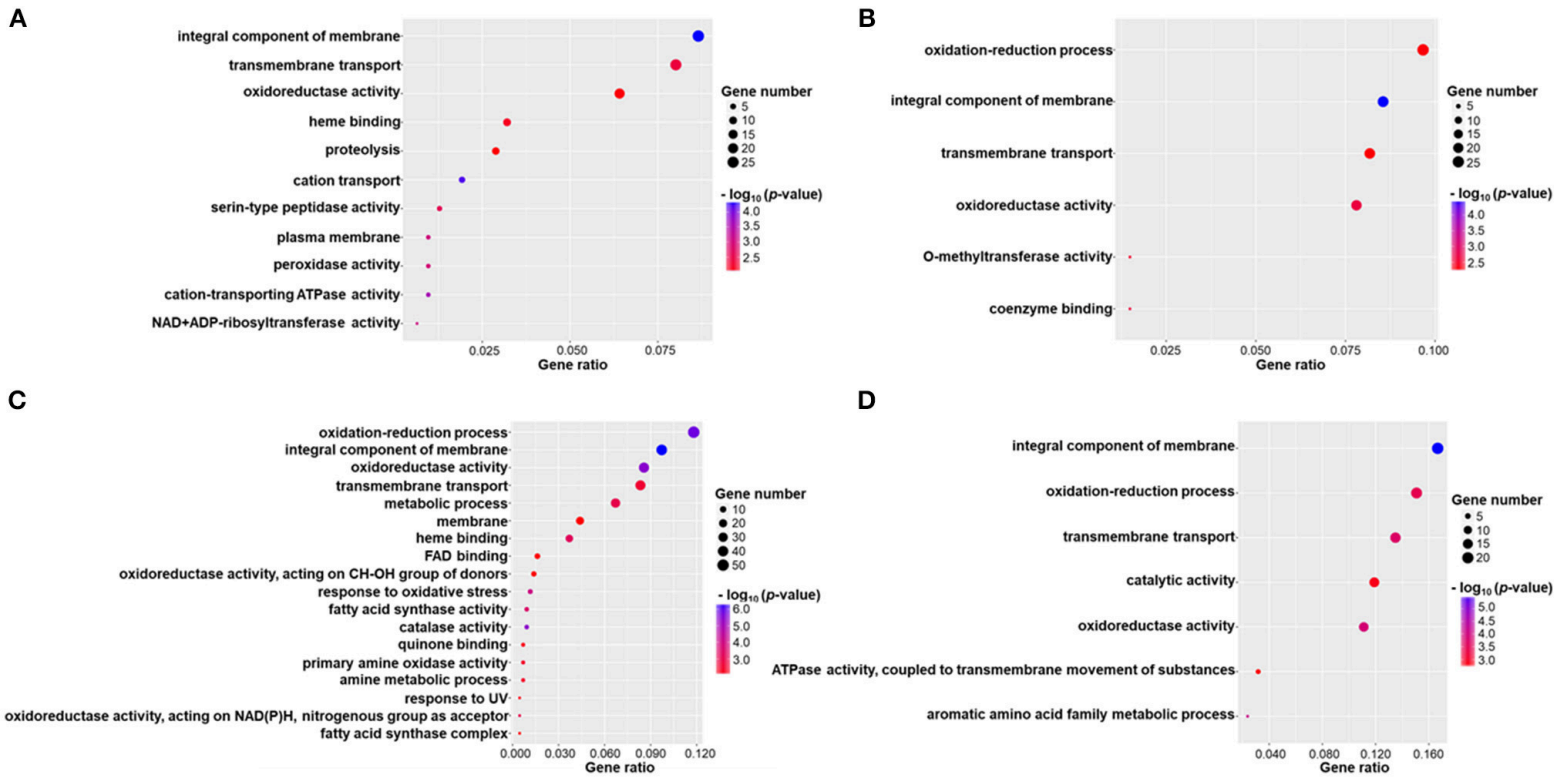
Gene ontology (GO) enrichment of differentially expressed genes (DEGs) in Δ*madsA* under four conditions. **(A–D)** Y, Y-to-M, M and M-to-Y conditions. The x-axis shows the gene ratio (the ratio of the gene count in GO terms to the total DEGs count) and the y-axis displays names of the enriched GO terms. M, mycelium; Y, yeast.

DEGs were successfully enriched and classified into three main categories: biological process, cellular component and molecular function. Compared with WT, cellular process, cell, cell part, binding, catalytic activity, and metabolic process were the most enriched GO terms in Δ*madsA* mutant. We identified 11, 6, 18, and 7 represented GO terms at Y, Y-to-M, M, and M-to-Y, respectively (Figure 8; Tables S4–S7). In yeast phase, GO terms of genes upregulated or downregulated included “integral component of membrane (GO:0016021),” “transmembrane transport (GO:0055085),” “cation transport (GO:0006812),” and “peroxidase activity (GO:0004601)” (Figure 8A). During Y-to-M transition, GO terms significantly enriched were those such as “oxidation-reduction process (GO:0055114)” and “integral component of membrane (GO:0016021)” (Figure 8B). For the mycelial phase, the represented GO terms included “response to oxidative stress (GO:0006979),” “metabolic process (GO:0008152)” and “response to UV (GO:0009411)” (Figure 8C). With regard to M-to-Y transition, “integral component of membrane (GO:0016021),” “oxidation-reduction process (GO:0055114)” and “transmembrane transport (GO:0055085)” were significantly enriched among the DEGs (Figure 8D). Of note, both in yeast and mycelial growth, the categories of upregulated genes included “transmembrane transport (GO:0055085)”. At all experimental conditions, the top five significantly enriched GO terms of genes upregulated or downregulated in Δ*madsA* mutant are shown in Table 2. Taken together, these data suggest that *madsA* is closely related to transmembrane transport, oxidation-reduction process, catabolic process and response to oxidative stress.

**Table 2:** Top 5 significantly enriched GO terms in Δ*madsA* genes upregulated or downregulated.

**Category**	**Y phase**	**Y-to-M transition**	**M-to-Y transition**	**M phase**
BP	GO:0006812 cation transport	GO:0055114 oxidation-reduction process	GO:0009072 aromatic amino acid family metabolic process	GO:0055114 oxidation-reduction process
			GO:0055085 transmembrane transport	GO:0006979 response to oxidative stress
			GO:0055114 oxidation-reduction process	
CC	GO:0016021 integral component of membrane	GO:0016021 integral component of membrane	GO:0016021 integral component of membrane	GO:0016021 integral component of membrane
	GO:0005886 plasma membrane			
MF	GO:0019829 cation-transporting ATPase activity	GO:0050662 coenzyme binding	GO:0016491 oxidoreductase activity	GO:0016491 oxidoreductase activity
	GO:0003950 NAD+ ADP-ribosyltransferase activity	GO:0016491 oxidoreductase activity		GO:0004096 catalase activity
		GO:0008171 O-methyltransferase activity		

*BP, biological process; CC, cellular component; M, mycelium; MF, molecular function; Y, yeast*.

### Verification of gene expression by RT-qPCR

The results obtained from the RNA-seq were validated by RT-qPCR with the same RNA samples. Several differently expressed genes including two genes (PMG0131830 and PMG0080490) related to oxidative tolerance were tested. Generally, the results of RT-qPCR were consistent with those of the RNA-seq (Table 3). For all the tested genes at different conditions, the trends of change were consistent in the two assays. The results suggest the reliability of the RNA-seq data. The expressions of two genes related to oxidative tolerance were found to be upregulated in both assays.

**Table 3:** Validation of the transcription levels of selected genes by RT-qPCR.

**Gene ID**	**Description**	**Phase**	**Fold change (S1/S2)*^a^***	**RT-qPCR*^b^***
PMG0062960	Integral membrane protein, putative	Y	10.76	2.41 ± 0.60^*^
PMG0022150	Cell surface glycoprotein 1	Y	0.15	0.24 ± 0.17^*^
PMG0062960	Integral membrane protein, putative	Y-to-M	8.30	5.79 ± 1.07^*^
PMG0030080	Demethylsterigmatocystin 6-O-methyltransferase	Y-to-M	0.00	0.02 ± 0.00^*^
PMG0600380	Protein rds1	M-to-Y	12.65	14.83 ± 8.02
PMG0180910	Hypothetical protein GQ26_0180910	M-to-Y	0.05	0.04 ± 0.02^*^
PMG0040630	Glutathione S-transferase Ure2-like, putative	M	14.04	24.62 ± 18.18
PMG0430050	Ent-kaurene oxidase	M	0.02	0.01 ± 0.01^*^
PMG0131830	Peroxisomal catalase	Y	2.65	2.42 ± 1.84^*^
PMG0080490	Catalase B	M	4.72	8.11 ± 5.64^*^

a *S1 and S2 indicated the FPKM value of a gene in ΔmadsA mutant and that gene in WT, respectively*.

b *RT-qPCR, relative transcription level of genes in ΔmadsA mutant compared with that of WT. Values are mean ± SD from three independent experiments*.

* *p < 0.05. FPKM, fragments per kilobase of transcript per million fragments mapped; M, mycelium; PCR, polymerase chain reaction; SD, standard deviation; WT, wild-type; Y, stable yeast*.

## Discussion

The MADS-box genes encode a eukaryotic family of transcriptional regulators involved in a number of important biological functions (Messenguy and Dubois, 2003). Previous studies revealed that the yeasts overexpressing *madsA* showed filamentous-like growth in *T. marneffei* at 37°C (Yang et al., 2014). This suggests that the *madsA* is involved in dimorphic transition. To better understand the roles of *madsA* in *T. marneffei*, its deletion mutant Δ*madsA* was obtained by homologous recombination in this study. Then the phenotypes and transcriptomes of Δ*madsA* and WT strains were compared at different growth conditions.

*T. marneffei* exhibits two distinct mycelium-yeast morphogenetic transitions and secretes the diffusible red pigment only at the mycelical phase, which could be used as a hallmark to indicate the transitions. For Y-to-M transition, *madsA* might play a regulatory role in this process. Deletion of *madsA* resulted in earlier occurrence of mycelium and red pigment, suggesting a short period to achieve the Y-to-M transition than those of WT and complemented strains. It is known that the pigments are composed of several compounds, in which some virulence factors have been identified in certain types of fungi (Noverr et al., 2004; da Silva et al., 2006; Silva et al., 2009). Thus, it would be interesting to study whether the *madsA* gene is involved in the virulence of *T. marneffei* in the future. In addition, the comparative transcriptomic analysis was conducted between the WT and Δ*madsA* strains. Based on the GO functional enrichment analysis, we found that the significant DEGs were mainly involved in transmembrane transport within the biological process group. It has been known that the iron and calcium transports were required for dimorphic switching (Campos et al., 2008; Hilty et al., 2011). The *madsA* may work by regulating genes related to iron or calcium transport. In another process, during M-to-Y transition, the phenotype of Δ*madsA* strain was identical to WT. As such, the *madsA* gene plays a key role in the transition of Y-to-M rather than M-to-Y. This is also supported by our observation that the expression level of *madsA* in WT is significantly higher in Y-to-M transition than that in M-to-Y transition.

In mycelial growth, we observed that the Δ*madsA* mutant produced much less conidia than WT at the initial stage. Another MADS-box transcription factor *VdMcm1* has been reported as a key regulator of conidiation of *Verticillium dahliae* (Xiong et al., 2016). The similar phenotypes were reported for two other genetic regulators of conidial development in *T. marneffei*, i.e., *yakA* and *rttA* (Suwunnakorn et al., 2014, 2015). The absence of *madsA* in *T. marneffei* also induces a delay in conidial germination in mycelial phase. Despite the low expression level of *madsA* in mycelial phase of WT, the absence of *madsA* caused numerous genes to be upregulated or downregulated. These suggest that *madsA* at low expression level in mycelium may still play important roles. The differences between Δ*madsA* and WT strains such as conidial production and germination in mycelium indirectly validated this conjecture.

In yeast phase, cells have to adapt to the reactive oxygen species (ROS) in the macrophage lysosome environment during host colonization. In *A. fumigatus*, the *rlmA* gene, a member of MADS-box transcription factors was reported to be involved in tolerance to oxidative damage and transcriptional regulation of genes related to oxidative stress adaptation (Rocha et al., 2016). In this study, we examined the response of the strains to H_2_O_2_, a ROS generated by macrophages. The deletion of *madsA* led to enhanced conidial tolerance to 25 mM H_2_O_2_ (Figure 4F). However, the chronic treatment of H_2_O_2_ at a concentration higher than 2.5 mM could inhibit the yeast and mycelial growth for both Δ*madsA* and WT. In addition, the GO term “response to oxidative stress (GO:0006979)” was found to be enriched among upregulated genes in Δ*madsA* mutant. Both RNA-seq and RT-qPCR data showed that two genes related to catalase (PMG0131830 and PMG0080490) were upregulated in both the yeast phase and the mycelial phase in the absence of H_2_O_2_ (*p* < 0.05, Table 3). However, in the presence of 2 mM H_2_O_2_, the expression level of PMG0131830 in yeast phase had no statistical difference between Δ*madsA* and WT strains; while the expression of PMG0080490 in the mycelial phase was downregulated in Δ*madsA* with 0.04-fold (*p* < 0.05). Our results indicated that the phenotypic and transcriptional patterns in response to oxidative stress seemed to be complicated. The methodological difference and other potential genes might affect the results and data interpretation. It is worth mentioning that the delay in germination might be a factor in the conidial tolerance to H_2_O_2_. Further investigations are needed to reveal the function of *madsA* in response to oxidative stress in *T. marneffei*.

In this study, it was important to choose a suitable housekeeping gene for transcriptional study. For *S. schenckii* and *P. brasiliensis*, it has been reported that the *act* gene had a differential expression during the dimorphic transition (Han-Yaku et al., 1996; Niño-Vega et al., 2007). However, a study compared the expression levels of the most commonly used housekeeping genes including *act, gapdh* and 18S rRNA gene across different cell types or under different conditions in *T. marneffei*, and validated that the *act* gene was the most suitable one for use as an endogenous control under morphological changes by RT-qPCR (Dankai et al., 2015). In addition, our results also showed that the threshold cycle (C_t_) values for the *act* gene in WT strain at four conditions (Y, M, Y-to-M and M-to-Y) were in the range of 20.12–20.76, while they were 20.42–20.82 for the Δ*madsA* mutant. There was no significant difference within strains under different conditions and between strains at the same condition (*p* > 0.05). Our results suggested that the *act* gene expression was stable in both WT and Δ*madsA* strains. Thus, the transcriptional analysis based on this should be reliable.

In a previous study we found that overexpression of *madsA* was able to induce mycelial growth at 37°C, at which temperature the WT strain grew as yeast cells (Yang et al., 2014). However, the results in this study showed that the Δ*madsA* mutant displayed a faster transition from yeast (37°C) to mycelium (25°C). The results from the two studies seemed to be contradictory. One reason might be that the overexpression strain was observed during continuous culture at 37°C in the previous study, while the typical phenotype of Δ*madsA* was observed at a temperature switching condition (from 37 to 25°C) in this study. As shown in Figures 6, 7, the DEGs profiles for Y and Y-to-M were different, suggesting that the roles of *madsA* should be temperature sensitive. In addition, genes other rather than *madsA* or in coupling with *madsA* might also contribute to transcriptional regulation of *T. marneffei* morphogenesis. It is also possible that a gene silencing process might be triggered by the overexpression of *madsA*. Further investigations on the MADS-box gene family and other potential genes are needed.

In summary, we have characterized Δ*madsA* and CMA strains of *T. marneffei* to reveal the potential role of *madsA* gene in morphogenesis, dimorphic transition and stress response. To sum up, *madsA* may be one of the key factors in the dimorphic transition. Moreover, our results provide evidence that *madsA* plays pleiotropic and global roles through regulating hundreds of genes. The DEGs affected by *madsA* will be further investigated. Its roles in the survival, pathogenicity and transmission of *T. marneffei* also call for more investigation. The data presented in this study would facilitate the understanding of transcription factor *madsA* in *T. marneffei*.

## Author contributions

EY, HZ, and TL conceived and designed the research, contributed reagents, materials, and supervised work. QW, SW, and LX carried out experiments. QW wrote the first draft of the manuscript. MD performed bioinformatics analysis and wrote sections of the manuscript. LL helped with assay set-up. LW gave critical review on the manuscript.

### Conflict of interest statement

The authors declare that the research was conducted in the absence of any commercial or financial relationships that could be construed as a potential conflict of interest. The reviewer GANV and handling editor declared their shared affiliation at time of review.

## Abbreviations

*act*: β-actin gene
*ble*: bleomycin-resistant gene
CMA: complemented strain
C_t_: threshold cycle
DEGs: differentially expressed genes
FPKM: fragments per kilobase of transcript per million fragments mapped
GO: gene ontology
H_2_O_2_: hydrogen peroxide
HIV: human immunodeficiency virus
*hph*: hygromycin B phosphotransferase gene
M: mycelium
PBS: phosphate buffered saline
PCR: polymerase chain reaction
RIN: RNA integrity numbers
ROS: reactive oxygen species
SD: standard deviation
SDA: sabouraud dextrose agar
SDB: sabouraud dextrose broth
SRA: sequence read archive database
WT: wild-type
Y: yeast.
