# Supplementary material for: MADS-Box Transcription Factor MadsA Regulates Dimorphic Transition, Conidiation, and Germination of Talaromyces marneffei
Source: Front Microbiol. 2018 Aug 7;9:1781. doi: 10.3389/fmicb.2018.01781 (PMC6090077; doi:10.3389/fmicb.2018.01781)
Supplement: Supplementary file 1 [file Table_1.DOCX]

**Table S1. The calculation for copy number of *hph* in Δ*madsA* mutant**

| **Gene** | **Mean of C_t_** | **ΔC_t_ = C_t (_*_actin_*_)_ - C_t (_*_hph_*_)_** | **2^-ΔCt^** | **Mean of 2^-ΔCt^** |
| --- | --- | --- | --- | --- |
| *act* | 23.440 |  |  |  |
| *hph* (primer pair 1) | 22.819 | 0.621 | 0.650 | 1.009 |
| *hph* (primer pair 2) | 23.227 | 0.213 | 0.863 | 1.009 |
| *hph* (primer pair 3) | 24.039 | -0.599 | 1.515 | 1.009 |

Three pairs of primers were designed for *hph* gene to avoid the influence of amplification efficiency. The single copied *act* gene was used as an endogenous control for analysis using the 2^(-ΔCT)^ method. *act*, β-actin gene; C_t_, threshold cycle; *hph*, hygromycin B phosphotransferase gene.
