# Supplementary material for: MADS-Box Transcription Factor MadsA Regulates Dimorphic Transition, Conidiation, and Germination of Talaromyces marneffei
Source: Front Microbiol. 2018 Aug 7;9:1781. doi: 10.3389/fmicb.2018.01781 (PMC6090077; doi:10.3389/fmicb.2018.01781)
Supplement: Supplementary file 2 [file Table_2.DOCX]

**Table S2. The quantity and quality of total RNA**

| **Sample name** | **Concentration (ng/μL)** | **Amount (μg)** | **OD260/280** | **Integrity (RIN)** |
| --- | --- | --- | --- | --- |
| Δ*madsA*-Y-1 | 620 | 37.2 | 2.16 | 9.5 |
| Δ*madsA*-Y-2 | 930 | 49.3 | 2.19 | 9.6 |
| Δ*madsA*-Y-3 | 585 | 31.0 | 2.14 | 9.9 |
| Δ*madsA*-M-1 | 273 | 16.4 | 2.19 | 9.3 |
| Δ*madsA*-M-2 | 114 | 6.0 | 2.19 | 9.3 |
| Δ*madsA*-M-3 | 326 | 17.3 | 2.16 | 9.5 |
| Δ*madsA*-Y-to-M-1 | 1,000 | 61.0 | 2.19 | 9.9 |
| Δ*madsA*-Y-to-M-2 | 862 | 45.7 | 2.20 | 9.8 |
| Δ*madsA*-Y-to-M-3 | 573 | 30.4 | 2.13 | 9.8 |
| Δ*madsA*-M-to-Y-1 | 107 | 6.3 | 2.16 | 8.8 |
| Δ*madsA*-M-to-Y-2 | 115 | 6.1 | 2.18 | 9.1 |
| Δ*madsA*-M-to-Y-3 | 119 | 6.3 | 2.13 | 9.4 |
| WT-Y-1 | 738 | 29.5 | 2.16 | 9.8 |
| WT-Y-2 | 217 | 11.5 | 2.17 | 9.6 |
| WT-Y-3 | 526 | 27.9 | 2.19 | 9.2 |
| WT-M-1 | 330 | 19.8 | 2.19 | 9.0 |
| WT-M-2 | 198 | 10.5 | 2.20 | 7.5 |
| WT-M-3 | 346 | 18.3 | 2.21 | 8.9 |
| WT-Y-to-M-1 | 1,040 | 62.4 | 2.16 | 9.3 |
| WT-Y-to-M-2 | 57 | 3.0 | 2.24 | 9.5 |
| WT-Y-to-M-3 | 585 | 31.0 | 2.18 | 9.3 |
| WT-M-to-Y-1 | 183 | 11.0 | 2.09 | 9.1 |
| WT-M-to-Y-2 | 60 | 3.2 | 2.22 | 8.3 |
| WT-M-to-Y-3 | 75 | 4.0 | 2.24 | 7.4 |

1, 2 and 3 represent the samples from three independent experiments, respectively. RIN, RNA integrity numbers; M, mycelium; WT, wild-type; Y, yeast.
