# Supplementary material for: MADS-Box Transcription Factor MadsA Regulates Dimorphic Transition, Conidiation, and Germination of Talaromyces marneffei
Source: Front Microbiol. 2018 Aug 7;9:1781. doi: 10.3389/fmicb.2018.01781 (PMC6090077; doi:10.3389/fmicb.2018.01781)
Supplement: Supplementary file 9 [file Data_Sheet_2.PDF]

Supplementary Material

MADS-Box Transcription Factor *MadsA* Regulates Dimorphic Transition, Conidiation, Germination of *Talaromyces marneffei*

Qiangyi Wang[1], Minghao Du[1,2], Shuai Wang[3], Linxia Liu[4,5], Liming Xiao[1,2], Linqi Wang[4], Tong Li[1,*], Hui Zhuang[1,*], Ence Yang[1,2,*]

[1] *Department of Microbiology & Infectious Disease Center, School of Basic Medical Sciences, Peking University Health Science Center, Beijing, 100191. P.R. China*
[2] *Institute of Systems Biomedicine, School of Basic Medical Sciences, Peking University Health Science Center, Beijing 100191, P. R. China*
[3] *Department of Laboratorial Science and Technology, School of Public Health, Peking University, Beijing 100191, P. R. China*
[4] *State Key Laboratory of Mycology, Institute of Microbiology, Chinese Academy of Sciences, Beijing, 100101. P.R. China*
[5] *University of Chinese Academy of Sciences, Beijing, 100049. P.R. China*

*Correspondence:
Tong Li
tongli08@vip.sina.com.
Hui Zhuang
zhuangbmu@126.com.
Ence Yang
yangence@pku.edu.cn.

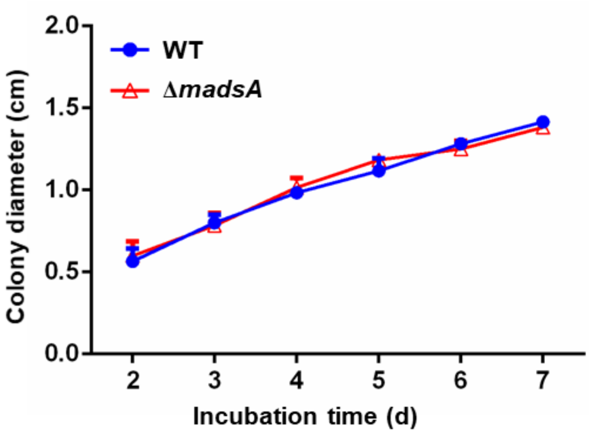

Supplementary Figure 2. Radial growth (colony diameter) of Δ*madsA* and WT strains in synchronizing culture experiment. The conidia of Δ*madsA* strain had a 4 h incubation in SDB prior to culture on SDA plates. SDA, Sabouraud Dextrose Agar; SDB, Sabouraud Dextrose Broth; WT, wild-type.
